# Efficacy of* Eucalyptus cinerea* as a Source of Bioactive Compounds for Curative Biocontrol of Crown Gall Caused by* Agrobacterium tumefaciens* Strain B6

**DOI:** 10.1155/2017/9308063

**Published:** 2017-07-03

**Authors:** Yosra Kahla, Karama Zouari-Bouassida, Fatma Rezgui, Mohamed Trigui, Slim Tounsi

**Affiliations:** ^1^Biopesticides Laboratory, Center of Biotechnology of Sfax, University of Sfax, P.O. Box 1177, 3018 Sfax, Tunisia; ^2^Analysis Department, Center of Biotechnology of Sfax, University of Sfax, P.O. Box 1177, 3018 Sfax, Tunisia

## Abstract

This research investigated the* Eucalyptus cinerea *leaves efficiency in the* Agrobacterium tumefaciens *biocontrol, the causative agent of crown gall. GC-MS analysis of the essential oil (EO) showed that the main components were 1,8-cineole (61%) and camphene (15.13%). Thanks to its polyphenols, flavonoids, quinones, terpenoids, alkaloids, and tannins richness, the EtOAc-F exhibited the most potent antibacterial activity in vitro. Indeed, compared to the other fractions, it has the lowest MIC and MBC values of 0.312 mg/mL and 2.5 mg/mL, respectively. The GC-MS analysis of EtOAc-F confirmed its richness in antibacterial compounds including gallic acid (7.18%), shikimic acid (5.07%), and catechin (3.12%). The time-kill curve assay of EtOAc-F (2.5 mg/mL) showed a potent bactericidal effect after 20 min of direct contact with* A. tumefaciens*. In planta experiments, gall weights were significantly reduced when EtOAc-F was applied at 0.625 and 2.5 mg/wounds. Besides, the disease reduction rates in gall weight were 95% and 97.5%, respectively. Interestingly, no phytotoxic effect was observed since tomato seeds germinated in the presence of the different concentrations of EtOAc-F. These results suggest that EtOAc-F has a good potential to be a curative biocontrol agent for crown gall disease.

## 1. Introduction

*Agrobacterium tumefaciens* is a gram-negative, rod-shaped, and soil-borne bacterium. In planta, infection by* A. tumefaciens* occurs through wounds in the roots or crown and at graft unions. This tumorigenic bacterium is the crown gall causal agent. This neoplastic disease appears throughout the world affecting nearly one thousand species of dicotyledonous [1]. Crown gall is a chronic disease that provokes severe damage on crops associated with important economic losses mainly in nurseries [2]. Plants with expanding galls may be unable to move water and nutrients up the trunk due to constricted or impaired vasculature and become weakened and unproductive and eventually die. Furthermore, infected plants are more likely to suffer from secondary infections and environmental stresses [3]. Prophylactic measures are the most used prevention approach [4]. Despite the precautions taken, the crown gall control is still very difficult because of its easy spread to other hosts.

The nonpathogenic strain* Agrobacterium radiobacter *K84 and the recombinant strain K1026 have been investigated and commercialized for their efficiency as a biological control of* A. tumefaciens *for many years [5–10]. However, K84 was reported to be efficient only against a few strains of* A. tumefaciens*. This failure is related to the transfer of resistance of the plasmid pAgK84 encoding the antibiotic bacteriocin K84 to the crown gall pathogens [11, 12]. In addition, the genetically modified strain K1026 is not certified for use in countries prohibiting genetically modified organism [13]. Consequently, the requirement for new antibacterial agents against* A. tumefaciens* has become greater than ever.

Previous studies highlighted that antimicrobial agents from medicinal plants have given a new alternative against resistant microorganisms [14, 15].* Eucalyptus* genus, belonging to* Myrtaceae* family, comprises about 900 species and originates from Australia [16]. Many studies revealed that* Eucalyptus* spp. have antimicrobial properties [17, 18]. It was reported that essential oil from* E*.* cinerea *also has antibacterial activity against Gram-positive and negative pathogenic bacteria [19, 20], but only a few reports dealing with the antibacterial activity against plant pathogenic bacteria were detailed.

To date and to the best of our knowledge, this is the first study that explores organic leaf fractions of* E*.* cinerea* for their antibacterial potential against* A. tumefaciens* to control crown gall disease in planta. Thus, we studied (i) the phytochemical analysis of* E. cinerea* leaf extracts and essential oil, (ii) the antibacterial effect of extracts and EO against* A. tumefaciens *in vitro, (iii) the mode of action, with reference to its phytochemical analysis by GC-MS, of the most active fraction, EtOAc-F, against* A. tumefaciens*, and (iv) the efficiency of the EtOAc-F for the biocontrol of crown gall in tomato plants.

## 2. Materials and Methods

### 2.1. Plant Material Collection

*E. cinerea* leaves were collected in January 2012 from Boulifa 36°07′25.7′′N  8°43′07.6′′E (Kef, Northwestern Tunisia). The sample was authenticated and a voucher specimen (LBPes EC 01.12) was deposited in the herbarium of the Laboratory of Biopesticides of the Centre of Biotechnology of Sfax.

### 2.2. Preparation of Essential Oil and GC-MS Conditions

The oil extraction was obtained from 1 kg of fresh* E. cinerea* leaves by steam distillation during 3 h using a “Clevenger” type apparatus. The aqueous phase was extracted with dichloromethane (3 × 50 mL) and dried with anhydrous sodium sulfate. The solvent was evaporated using the Rotavapor to afford 3.6 g of the essential oil (EO) which was stored at 4°C prior to further analyses. The EO was solubilized in n-hexane for chromatographic analysis coupled with mass spectrometry.

This EO was performed with GC 6890N and 5975B MS Agilent model, equipped with an Agilent Technologies capillary HP-5MS column (30 m × 0.25 mm i.d. × 0.25 *μ*m film thickness) and an electron impact ionization (ionization voltage 70 eV; all Agilent, Santa Clara, CA). The carrier gas was helium used at 1 mL/min flow rate. The oven temperature program started from 35°C (held for 3 min) and then was programmed to rise to 250°C at a rate of 5°C/min. The chromatograph was equipped with a split/splitless injector used in the splitless mode.

EO components were identified by comparing their Kovats index and mass spectral fragmentation patterns with those of the standards stored on the Wiley Registry of Mass Spectral Data 7th edition (Agilent Technologies, Inc.) and National Institute of Standards and Technology 05 MS (NIST) library data.

### 2.3. Plant Extracts Preparation

The leaves were washed with distilled water and dried in the shade. Then, they were crushed to a fine powder and the resulting material (100 g of powder) was extracted by hydroalcoholic maceration in ethanol-water 80% with occasional shaking, at room temperature. The dried hydroethanolic crude extract (20 g) was suspended in 200 mL distilled water and was sequentially partitioned into solvents with increasing polarity: hexane (3 × 300 mL), ethyl acetate (3 × 300 mL), and butanol (3 × 300 mL). The filtered solution was evaporated at reduced pressure (Rotary Evaporator Buchi R-200, Switzerland) and the remaining aqueous layer was lyophilized to give the water fraction. Four fractions of* E. cinerea *were obtained: hexanic fraction (Hex-F), ethyl acetate fraction (EtOAc-F), butanolic fraction (ButOH-F), and water fraction of* E. cinerea* (W-F). The stock solutions were kept at 4°C in the dark until further analysis.

#### 2.3.1. Preliminary Qualitative Analysis

The qualitative phytochemical tests were performed according to Allen [21] and Harborne [22]. They were based on the visual observation of color change of* E. cinerea* fractions. The chemical constituents tested are phenolics, flavonoids, quinones, terpenoids alkaloids, and tannins.

#### 2.3.2. Determination of Total Phenolic Content

The total phenolic content was determined using the Folin–Ciocalteu method adapted to a microscale described by Waterman and Mole [23]. Gallic acid was used as a standard. The absorbance was measured at 760 nm and the phenolic contents are expressed in mg of gallic acid equivalent per g of dry plant extract (mg GAE/g).

#### 2.3.3. Determination of Total Flavonoids Content

The flavonoids content in fractions was determined spectrophotometrically according to Quettier-Deleu et al. [24]. The absorbance was measured at 430 nm and the flavonoids content was expressed in mg of Quercetin equivalent per g of dry plant extract (mg QE/g).

### 2.4. Investigation of the Antibacterial Activity In Vitro

#### 2.4.1. Microorganisms

The tested plant pathogenic bacterium was* A. tumefaciens* strains B6. It was kindly provided by the Olive Institute of Sfax, Tunisia.* A. tumefaciens* B6 was cultivated in Mannitol Glutamate Agar (MGA) containing 5 g/l D-mannitol, 2 g/l L-glutamic acid, 0.5 g/l KH2PO4, 0.2 g/l MgSO4.7H2O, and 20 g/l agar, pH 7.2, at 30°C for 48 h.

#### 2.4.2. Agar Diffusion Method and Determination of MIC and MBC

The* E. cinerea* leaf extracts antibacterial activity was evaluated by means of agar-well diffusion assay according to Güven et al. [25]. Minimum Inhibitory Concentrations (MICs) were determined according to Eloff [26] with minor modifications. The test was performed in sterile 96-well microplates with a final volume in each microplate well of 100 *μ*l. For susceptibility testing, 100 *μ*l of MG was distributed from the second to the final wells. The first well of the microplate was prepared by dispensing 180 *μ*l of the growth medium and 10 *μ*l of the different extracts to reach a final concentration of 10 mg/mL; 90 *μ*l of scalar dilutions was then transferred from the second to the final well. Finally, 10 *μ*l of the bacterial suspensions (10^6^ CFU/mL) was added. The plates were incubated at 30°C for 48 h. The MIC was defined as the lowest concentration of the total extract at which the microorganism does not demonstrate visible growth after incubation. The 3-(4,5-Dimethyl-2-thiazolyl)-2,5-diphenyl-2H-tetrazolium bromide (MTT) was used as an indicator of microorganism growth (25 *μ*l was added to each well). Where microbial growth was inhibited, the solution in the well remained clear after incubation with MTT. The Minimum Bactericidal Concentrations (MBCs) were determined by serial subcultivation of 5 *μ*l from each well that showed no color in MG plates after incubation for 48 h at 30°C. The lowest concentration with no visible growth was defined as the MBC, indicating that >99.9% of the original inoculum was killed.

#### 2.4.3. GC-MS Analysis of* E. cinerea* EtOAc-F

Twenty mg of EtOAc-F was trimethylsilylated using 50 *μ*L of N,O-bis(trimethylsilyl)trifluoroacetamide (BSTFA) and 50 *μ*L of pyridine at 60°C for 1 hour and then analyzed by GC-MS. The GC oven temperature was held at 100°C for 1 min and was then programmed to go from 100 to 260°C at a rate of 4°C/min and then, it held for 10 min. The split/splitless injector (splitless mode) temperature was set at 280°C. The components were identified by careful examination of fragmentation patterns and the spectral data obtained from the Wiley and NIST libraries.

#### 2.4.4. Time-Kill Assay of EtOAc-F on* A. tumefaciens*

The EtOAc-F effect on the survival of* A. tumefaciens* was evaluated using the viable cell count procedure in a physiological saline solution according to the method of Bajpai et al. [27] with some modifications. Active cultures for viable count assay were prepared in MG, grown at 30°C for 48 h. For each strain, 1 mL of active stock solution (approximately 10^9^ CFU/mL) was transferred to 2 mL of Eppendorf tube. The cultures were then centrifuged at 10,000 rpm for 10 min. The pellets were retained and resuspended with 1 mL of phosphate-buffered saline (PBS). For viable counts, each of the tubes containing resuspended bacterial suspension (approximately 10^9^ CFU/mL) of B6 was inoculated with 100 mL of EtOAc-F fraction at 2MIC (0.625 mg/mL) and CMB (2.5 mg/mL) concentration in 900 mL. Samples for viable cell counts were taken out at 0, 10, 20, 30, 40, 50, and 60 min time intervals. The viable plate counts were monitored as follows: after incubation, 100 mL of the resuspended culture was diluted into 900 mL PBS (10-fold). Then, a 100 mL sample of each treatment was diluted and spread on the surface of MG agar. The colonies were counted after 48 h of incubation at 30°C. The controls were inoculated without EtOAc-F and with the same experimental condition as mentioned above. Each assay in this experiment was replicated three times.

### 2.5. Germination Test

Tomato (Rio Grande) seeds were surface sterilized for 20 min on a 5% (v/v) NaCl solution and rinsed several times with sterile distilled water. The seeds were then soaked and mixed with sterile distilled water for 2 h. Germination assays of tomato seeds were carried out by placing seeds treated with 2MIC, MBC, and 2MBC (0.625, 2.5, and 5 mg/mL, resp.) of EtOAc-F in Petri dishes with filter paper. As a control, the seeds were germinated in distilled water. The tomato seeds were incubated in darkness in a growth chamber at 27°C for 7 days. The experiments were conducted in a completely randomized design, with three replicates per treatment (10 seeds per dish). The seeds were considered to have germinated as soon as the radicle pierced the envelope.

### 2.6. Suppression of Crown Gall Disease in Pot Experiments

For this in vivo test, one-month-old tomato plants (cv. Rio Grande) were used. They were grown in pots (15 cm diameter) containing a sterilized peat and watered daily. The pathogenicity of* A. tumefaciens* B6 was verified by tested induced galls on tomato plants 21 days after stem inoculation [28]. To explore the effect of suppression of crown gall disease, firstly, 10 *μ*l of suspension of* A. tumefaciens* (10^9^ CFU/mL) was inoculated on 1 cm long longitudinal wounds made with a sterile scalpel at the internodes (30 wounds per treatment). Then, after two hours, EtOAc-F was added to wounds at different concentrations (0.625, 2.5, and 4 mg/wounds). Wounds were covered with parafilm to prevent drying. Plants inoculated only with bacterium served as positive controls. After three weeks, the galls weight was determined. The crown gall disease severity was estimated as average of gall weight g/plant. Percentage of disease reduction (PDR) was calculated from weight of galls [29, 30] as follows:

(1) $$\begin{array}{c} PDR=\frac{C-E}{E}\times 100, \end{array}$$

where *C* is average of gall weight in control treatment. *E* is average of gall weight in treatments.

### 2.7. Statistical Analysis

All the data were expressed as mean values ± standard deviation. Statistical comparisons were carried out using GraphPad prism 6, analyzed by one-way ANOVA, followed by Tukey's post hoc test for multiple comparisons with statistical significance.

## 3. Results and Discussion

### 3.1. GC-MS Analysis of* E. cinerea* Essential Oil

The GC-MS analysis of the EO (Table 1) led to the identification of 32 compounds representing 98.64% of the oil. The analysis revealed a complex mixture of EO consisting mainly of oxygenated monoterpenes and monoterpene hydrocarbons followed by oxygenated sesquiterpenes and sesquiterpene hydrocarbons. As shown in Table 1, the major components of the EO were identified to be 1,8-cineole (61%) and camphene (15.13%) beside other constituents with relatively low concentrations including *α*-terpineol (4.77%), globulol (4.06%), *α*-pinene (3.45%), trans-pinocarveol (2.98%), aromadendrene (1.15%), and 4-terpineol (1.02%). These results showed relative differences in composition from that of the EO derived from the same species from Ain Draham, Tunisia [20]. Indeed, it was reported that the major components were 1,8-cineole (70.7%) and *α*-terpineol (10.7%), while camphene was not detected [20]. This variation might be due to the effect of climatic and geographical factors and harvesting season.

### 3.2. Phytochemicals Analysis of Organic Extracts

The preliminary phytochemical screening of hydroethanolic extract of* E. cinerea* leaves and its fractions indicated that polyphenols, flavonoids, quinones, terpenoids, alkaloids, and tannins are more abundant in the EtOAc-F (Table 2). Moreover, the quantitative estimation of the total phenolic contents (Table 3) showed that the EtOAc-F contains the highest amount of phenols (70.09 mg GAE/g) followed by the ButOH-F and EtOH-H2OE ones (62.07 and 59.25 mg GAE/g, resp.). The EtOH-H2OE and EtOAc-F have the highest total flavonoid contents (16.74 and 12.27 mg QE/g, resp.) followed by the ButOH-F and W-F (6.58 and 0.27 mg QE/g).

These secondary metabolites showed differences in their contents in terms of solvents polarities and therefore their solubility which depends on their structures and polymerization degree [31].

### 3.3. In Vitro Antibacterial Effect of* E. cinerea* against* A. tumefaciens*

The antibacterial activity of the hydroethanolic extract, organic fractions, and EO of* E. cinerea *was quantitatively evaluated by measuring the diameter of the inhibition zone and the determination of the MIC and MBC. The activity was examined against* A. tumefaciens*, a gram-negative phytopathogenic bacterium and the causative agent of crown gall. The obtained results are summarized in Table 4. The best antibacterial activity against* A. tumefaciens* was achieved with EtOAc-F; in fact, MIC and MBC recorded the lowest values of 0.312 and 2.5 mg/mL, respectively. This was expected since previous studies showed that polyphenols, flavonoids, alkaloids, and tannins are active against pathogenic and phytopathogenic bacteria [32–34]. The ButOH-F and EO showed modest activities. This could be due mainly to the weakness of ButOH-F and EO in active anti-*Agrobacterium* phytocompounds [35]. The EtOH-H_2_OE, Hex-F, and W-F were inactive at the tested concentrations. According to the previously mentioned results, EtOAc-F was chosen for further investigation.

### 3.4. GC-MS Analysis of* E. cinerea* EtOAc-F

The identification of the constituents of the EtOAc-F with GC-MS and their retention time, their content (%), and their characteristic fragments are listed in Table 5. Nine phenolic components were identified in EtOAc-F with a content of 20.54%. The most abundant ones were gallic acid (7.18%), shikimic acid (5.07%), catechin (3.12%), 2-(diphenylphosphoryl)-4-nitrophenol (2.74%), and protocatechuic acid (1.13%). Three terpenes were detected: camphene (2.11%), *α*-gurjunene (2.05%), and aromadendrene (1.08%). This fraction also contains some sugars including galactopyranose (3.62%), D-mannopyranose (2.48%), *α*-D-glucopyranoside (0.75%), and *β*-D-galactofuranose (0.67%). Besides, the EtOAc-F harbors several other components including palmitic, stearic, and oleic acids phytol and inositol. Phenolic compounds, reported in EtOAc-F, are known for their biological activities and beneficial effects [24].

To the best of our knowledge, this work is the first attempt to investigate the phytochemical composition of* E. cinerea* organic extracts by GC-MS and to analyze the relationship between its chemical composition and antitumor activity against* A. tumefaciens*.

### 3.5. Time-Kill Curve Assay of EtOAc-F on* A. tumefaciens*

**Figure 1:**
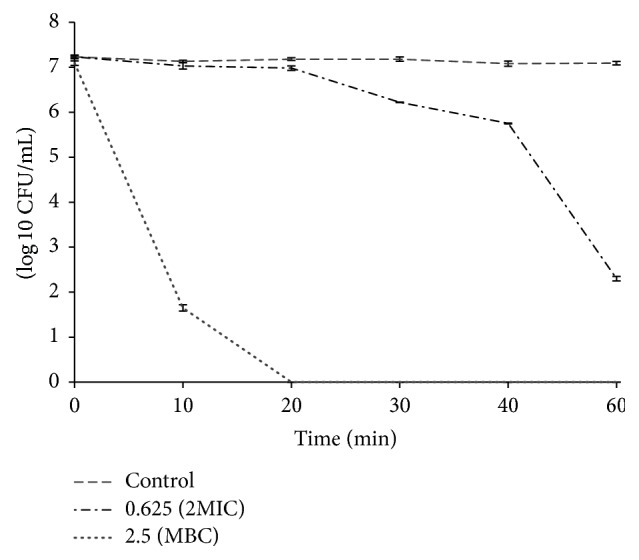
Time-kill curves of* A. tumefaciens* treated with different concentrations (0.625 and 2.5 mg/mL) of EtOAc-F. Samples were taken at different incubation times and viability was determined by the plate colony count procedure (CFU: Colony Forming Unit).

In order to determine the EtOAc-F mode of action (bacteriostatic or bactericidal) on* A. tumefaciens*, a time-kill curve experiment was carried out using two concentrations, 0.625 and 2.5 mg/mL. At 0.625 mg/mL EtOAc-F showed bacteriostatic activity. A bactericidal effect was recorded at 2.5 mg/mL after 20 min of contact time (Figure 1). This bactericidal effect could be due to the richness of the EtOAc-F in phenolic compounds such as gallic, shikimic, protocatechuic, gentisic, caffeic, and ferulic acids and catechin. These can inhibit the enzymes and the bacteria nucleic acids and interact with the cytoplasmic membrane promoting its destabilization and permeabilization [36–38]. These bioactive compounds can act individually or synergistically to induce bacterial death. Mhalla et al. [31] reported that the use of ethyl acetate fraction from* Rumex tingitanus* at a concentration of 1.25 mg/mL and 2.5 mg/mL caused a bactericidal activity after 20 and 10 min, respectively, against the foodborne pathogens* Listeria monocytogenes*. Knezevic et al. [39] used time-kill curves analysis to reveal the synergistic interaction between* E. camaldulensis* essential oil and polymyxin B which reduced bacterial count* Acinetobacter baumannii* after 6 h of incubation.

### 3.6. Germination Test

**Figure 2:**
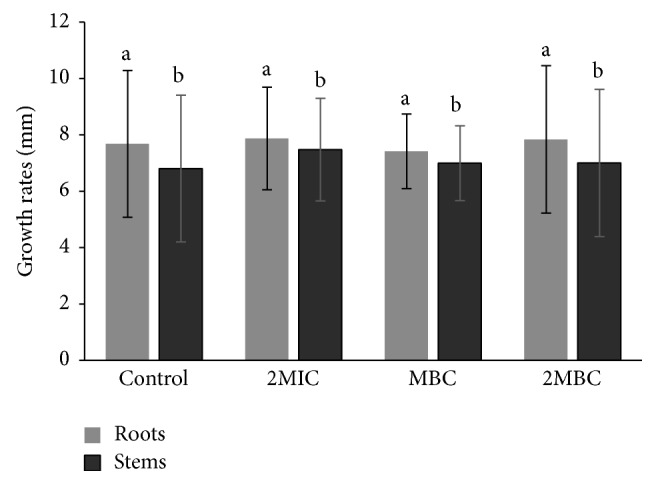
Effect of* E. cinerea *EtOAc-F on seedling growth of tomato roots and stems. Data are expressed as mean ± SD for rates of roots of 30 seeds in each group. Means followed by the same letters are not significantly different at *P* > 0.05.

In order to study the EtOAc-F phytotoxicity, tomato seeds were incubated with different concentrations (0.625, 2.5, and 5 mg/mL that correspond to 2MIC, MBC, and 2MBC, resp.) (Figure 2). The obtained results showed that 100% of the seeds germinated after adding different concentrations of the fraction. Moreover, no significant differences were observed in tomato growth rates of roots and stems in the presence of the used concentrations (Figure 2). The EtOAc-F does not seem to be phytotoxic. Contrarily to EtOAc-F, Grichi et al. [40] reported that the essential oil of* E. cinerea* (at 0.14–0.35 mg/mL) was shown to be phytotoxic against* Sinapis arvensis, Erica vesicaria, Scorpiurus muricatus, Triticum durum, Vicia faba, and Phaseolus vulgaris*. It reduced emergence, as well as seedling growth and root and shoot length. Similarly, essential oil from* E. citriodora *reduced seedling growth and dry weight accumulation in* Cassia occidentalis, Amaranthus viridis, *and* Echinochloa crusgalli *[41]. Saeed et al. [42] reported that* E. camaldulensis *leaf aqueous extracts used at 5, 10, and 15% affect germination and seedling growth of* Datura *spp. and* Sinapis *spp. but not those of* Sonchus *spp.

In our case, the lack of inhibitory action of EtOAc-F on seed germination even at the highest concentration may suggest that its bioactive compounds do not display a high allelopathic potential [43]. Different studies reported that an extract of plant could act as stimulator and be phytotoxic (inhibitor) at the same time depending on the response of the plant seeds, the concentration, and interaction of its secondary metabolites with the edaphic parameters [43–46]. Moreover, allelopathicity may vary among plant parts depending on the tissue and extract types [47].

### 3.7. Suppression of Crown Gall Disease in Pot Experiment

**Figure 3:**
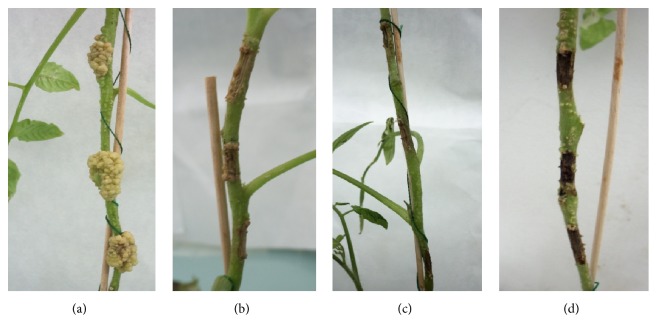
Effect of* E. cinerea *EtOAc-F on the inhibition of excrescences induced 21 days after inoculation with* A. tumefaciens.* (a) Control not treated (10^9^ CFU/mL), (b) tomato treated with concentration of 0.625 mg/wound, (c) tomato treated with concentration of 2.5 mg/wound, and (d) tomato treated with concentration of 4 mg/wound.

In order to evaluate the effect of EtOAc-F on the biocontrol of* A. tumefaciens* B6, tomato plants were treated with different concentrations (0.625, 2.5, and 4 mg/wounds). During the test, normal growth of the tomato seedlings was observed. Results observed in Figure 3 indicated that all tested concentrations of EtOAc-F significantly reduced the symptoms of tomato crown gall, compared to control plants. Indeed, untreated plants showed well-developed tumors that occupy all the site of the wounds. Tumors present an irregular, whitish, and spongy aspect. However, plants treated with 0.625 mg/w showed very small tumors, separated and installed at the extremities of wounds. Insignificant galls were observed for plants treated with 2.5 and no galls were observed after treatment with 4 mg/w. These findings were confirmed by the determination of gall weights and the percentage of disease reduction (Table 6). Data show that the average weight of crown gall decreased highly significantly after adding increased concentrations of EtOAc-F in comparison with the control. The lowest average weight of galls (0.01 g) was obtained when the fraction was used at a concentration of 2.5 mg/w and the percentage of the disease reduction in gall weight was 97.5%, whereas the average of gall weight of the untreated plants was 0.4 g.

Moreover, the statistical analysis showed that the difference in gall weight after using the tested concentrations is not significant, showing the efficiency of 0.625 mg/w concentration in reducing disease severity (95%). In accordance with our study, Trigui et al. [32] showed that the EtOAc-F of* Lawsonia inermis *significantly inhibited the formation of crown gall on tomato plants. However, Ashraf et al. [48] reported that methanol extract from* E. camaldulensis *leaves exhibited a potent activity in suppressing gall formation induced by* A. tumefaciens *on potato disc. Thus, the nature of the extraction solvents has a strong influence on the profile of the bioactive compounds and their particular activity spectrum. Gallic, shikimic, and protocatechuic acids and catechin were proven to possess a potential antitumor activity. Therefore, the efficiency of EtOAc-F may be especially due to its richness of such compounds. Ho et al. [49] reported that gallic acid exhibits strong anticancer properties, counting cytotoxic effects and inhibition of cell migration over the suppression of some of signaling pathways. Catechin was found to have an anti-invasive activity that can be attributed to its capacity to bind to extracellular matrix and to inactivate a number of enzymes [50]. Tseng et al. [51] revealed that protocatechuic acid extracted from* Hibiscus sabdariffa* exhibited an antiproliferative effect against cancer cells by inducing apoptosis.

Several studies reported that the mechanism of tumor induction between human and plant pathogens* A. tumefaciens* is similar and shares the same strategy. As the EtOAc-F is efficient against the neoplastic disease crown gall, it could be a potential extract to develop tumor treatment in human beings.

## 4. Conclusion

This work provided novel information about the antitumoral activity of* E. cinerea*. The analysis of the most active extract (EtOAc-F) by GC-MS revealed the presence of nine phenolic compounds known for their strong antibacterial activity. Moreover, an inhibition of crown gall disease by the EtOAc-F was observed after bacterial inoculation, suggesting that this fraction had both protective and curative effects against the soil-borne plant pathogen* A. tumefaciens*. The possibility of controlling crown gall disease with* E. cinerea* seems of particular interest considering the unavailability of commercial cultivars resistant to* A. tumefaciens*. Further tests are required to confirm our results for the exploitation of this fraction as a source for new anticancer drugs in human beings.

## Figures and Tables

**Table 1:** Chemical composition of essential oil isolated from *E. cinerea* leaves.

Compounds^a^	Area (%)^b^	KI^c^
*Monoterpene hydrocarbons*		
*α*-Pinene	3.45	941
Camphene	15.13	953
*β*-Pinene	0.17	978
3-Carene	0.06	1004
*α*-Terpinene	0.17	1015
*γ*-Terpinene	0.11	1065
Terpinolene	0.04	1088
Bornylene	0.11	1232
*Oxygenated monoterpenes*		
1,8-Cineole	61.00	1033
Fenchol	0.11	1117
trans-Pinocarveol	2.98	1139
Pinocarvone	0.92	1165
4-Terpineol	1.02	1181
*α*-Terpineol	4.77	1196
trans-Carveol	0.36	1217
l-Bornyl acetate	0.09	1285
exo-2-Hydroxycineole acetate	0.22	1367
cis-Jasmone	0.05	1398
*Sesquiterpene hydrocarbons*		
*α*-Gurjunene	0.01	1407
*β*-Caryophyllene	0.02	1418
*β*-Gurjunene	0.04	1440
Aromadendrene	1.15	1456
*β*-Guaiene	0.07	1459
*β*-Selinene	0.04	1480
*α*-Selinene	0.02	1488
Bicyclogermacrene	0.14	1494
*γ*-Cadinene	0.09	1512
*Oxygenated sesquiterpenes *		
Epiglobulol	0.69	1561
Spathulenol	0.06	1576
Globulol	4.06	1582
Viridiflorol	1.41	1612
*Hydrocarbons*		
6,7-Dimethyltetralin	0.08	1393

*Total identified compounds*	98.64%	
*Total monoterpene hydrocarbons*	19.24	
*Total oxygenated monoterpenes*	71.52	
*Total sesquiterpene hydrocarbons*	1.58	
*Total oxygenated sesquiterpenes*	6.22	

^a^Identification of components based on GC-MS Wiley 7.0 version library and National Institute of Standards and Technology 05 MS (NIST) library data. ^b^Percentages area. ^c^KI: Kovats indices on HP-5MS capillary column.

**Table 2:** Preliminary phytochemical screening.

	EtOH-H_2_OE	Hex-F	EtOAc-F	ButOH-F	W-F
Polyphenols	++	−	++	++	+
Flavonoids	++	−	++	+	−
Quinones	+	−	+	+	−
Terpenoids	+	−	++	+	+
Alkaloids	+	−	++	+	+
Tannins	+	−	++	+	+

(++) Abundant, (+) present, and (−) absent; EtOH-H_2_OE: hydroethanolic extract of *E. cinerea* leaves; Hex-F: hexane fraction of *E. cinerea*; EtOAc-F: ethyl acetate fraction of *E. cinerea*; ButOH-F: butanol fraction of *E. cinerea*; W-F: water fraction of *E. cinerea*.

**Table 3:** Total phenolic and flavonoid contents of *E. cinerea* extracts.

Fractions	EtOH-H_2_OE	Hex-F	EtOAc-F	ButOH-F	W-F
TPC (mg GAE/g)	59.25 ± 0.38	nd	70.09 ± 0.08	62.07 ± 0.48	55.2 ± 0.14
TF (mg EQ/g)	16.74 ± 0.35	nd	12.27 ± 0.01	6.58 ± 0.01	0.27 ± 0.01

TPC (mg GAE/g): mg of gallic acid equivalent per g of dry plant extract; TF (mg QE/g): mg of Quercetin equivalent per g of dry plant extract; nd: not detected; each value represents the mean ± SD of three experiments.

**Table 4:** Antibacterial activity of *E. cinerea *extracts and EO and determination of the Minimum Inhibitory Concentrations (MICs) and Minimum Bactericidal Concentrations (MBCs).

	Inhibition zones diameter (mm)^a^	MIC (mg/mL)	MBC (mg/mL)
EtOH-H_2_OE	0	—	—
Hex-F	0	—	—
EtOAc-F	15.33 ± 0.58	0.312	2.5
ButOH-F	11.5 ± 0.5	2.5	>10
W-F	0	—	—
EO	11 ± 0.87	10	>10

Values are given as mean ± SD of triplicate experiment. ^a^Diameter of inhibition zones of *E. cinerea *fractions including diameter of disc 8 mm; —: not tested.

**Table 5:** GC-MS analysis of *E. cinerea *ethyl acetate fraction (EtOAc-F).

Compounds	*t*_*R*_ (min)	Content (%)	Characteristic mass fragments
*Phenolic compounds*			
4-Hydroxybenzoate	15.97	0.23	282, 267, 193, 223, 73
Gentisic acid	20.100	0.68	370, 355, 281, 147, 223, 267, 73
Protocatechuic acid	21.140	1.13	147, 223, 355, 311, 281, 193, 73
Shikimic acid	21.345	5.07	174, 179, 281, 311, 355, 443, 458, 73
Gallic acid	24.845	7.18	458, 281, 443, 355, 399, 179, 147, 73
2-(Diphenylphosphoryl)-4-nitrophenol	25.606	2.74	115, 139, 183, 215, 292, 339
Ferulic acid	27.48	0,2	146, 191, 219, 249, 308, 323, 338, 73
Caffeic acid	28.571	0.19	219, 381, 396, 73
Catechin	43.889	3.12	179, 368, 650, 267, 355, 73
*Terpenes*			
Camphene	8.700	2.11	79, 93, 107, 121, 136
Aromadendrene	10.947	1.08	41, 55, 69, 107, 121, 134, 147, 161, 175, 189, 204
*α*-Gurjunene	19.646	2.05	41, 55, 77, 91, 105, 119, 133, 147, 161, 189, 204
*Sugars*			
*β*-D-Galactofuranose	22.370	0.67	103, 147, 189, 217, 319, 73
Galactopyranose	23.475	3.62	103, 147, 204, 249, 307, 331, 73
D-Mannopyranose	25.796	2.48	103, 135, 147, 204, 249, 307, 331, 73
*α*-D-Glucopyranoside	39.562	0.75	103, 135, 147, 217, 271, 319, 361, 437, 73
*Others*			
Phosphoglycerol	20.210	0.62	103, 218, 299, 318, 357, 387, 445, 73
Palmitic acid	26.338	1.26	117, 145, 129, 132, 313, 73
Inositol	27.978	0.66	147, 191, 205, 217, 265, 306, 318, 73
Phytol	29.201	0.36	123, 143, 103, 73
Oleic acid	30.190	0.75	117, 129, 145, 185, 222, 264, 339, 73
Stearic acid	30.614	0.76	117, 147, 201, 297, 341, 423, 73
1,2,4,8-Tetramethylbicyclo[6.3.0]undeca-2,4-diene	31.332	0.11	109, 147, 204, 219, 73
1H-Cycloprop[e]azulene	33.367	0.23	147, 204, 247, 287, 575, 73
Cholest-5-en-3-ol	45.434	0.04	147, 217, 283, 368, 456, 73

*t*_*R*_: retention time.

**Table 6:** Efficacy of different concentrations of *E. cinerea *ethyl acetate fraction (EtOAc-F) in suppression of tomato gall formation induced by *A. tumefaciens* B6.

Treatment (mg/w)	Number of analyzed plants	Average of gall weight (g)	Reduction of gall weight (%)
Control (untreated)	10	0.4 ± 0.2^a^	0
0.625	10	0.02 ± 0.02^b^	95
2.5	10	0.01 ± 0.01^b^	97.5
4	10	0	100

Data are expressed as mean ± SD for average of gall weights of 30 galls in each group. Significant differences were observed between control and treated groups: ^a^*P* ≤ 0.0001; no significant differences were observed between treated groups: ^b^*P* > 0.05.
